# Turning up the volume on mutational pressure: Is more of a good thing always better? (A case study of HIV-1 Vif and APOBEC3)

**DOI:** 10.1186/1742-4690-5-26

**Published:** 2008-03-13

**Authors:** Satish K Pillai, Joseph K Wong, Jason D Barbour

**Affiliations:** 1 Department of Medicine, University of California, San Francisco, CA 94121, USA; 2 Veterans Affairs Medical Center, San Francisco, CA 94121, USA

## Abstract

APOBEC3G and APOBEC3F are human cytidine deaminases that serve as innate antiviral defense mechanisms primarily by introducing C-to-U changes in the minus strand DNA of retroviruses during replication (resulting in G-to-A mutations in the genomic sense strand sequence). The HIV-1 Vif protein counteracts this defense by promoting the proteolytic degradation of APOBEC3G and APOBEC3F in the host cell. In the absence of Vif expression, APOBEC3 is incorporated into HIV-1 virions and the viral genome undergoes extensive G-to-A mutation, or "hypermutation", typically rendering it non-viable within a single replicative cycle. Consequently, Vif is emerging as an attractive target for pharmacological intervention and therapeutic vaccination. Although a highly effective Vif inhibitor may result in mutational meltdown of the viral quasispecies, a partially effective Vif inhibitor may accelerate the evolution of drug resistance and immune escape due to the codon structure and recombinogenic nature of HIV-1. This hypothesis rests on two principal assumptions which are supported by experimental evidence: a) there is a dose response between intracellular APOBEC concentration and degree of viral hypermutation, and, b) HIV-1 can tolerate an elevated mutation rate, and a true error or extinction threshold is as yet undetermined. Rigorous testing of this hypothesis will have timely and critical implications for the therapeutic management of HIV/AIDS, and delve into the complexities underlying the induction of lethal mutagenesis in a viral pathogen.

## Commentary

The evolutionary potential of HIV-1 is unquestionably one of the key factors underlying its extreme resilience in the face of host immunity and antiretroviral drug pressure. RNA viruses in general have high mutation rates, and HIV-1 is not an exception with an estimated rate of 3.4 × 10^-5 ^mutations/site/generation, owing to the poor fidelity of reverse transcriptase and a lack of proofreading machinery [1]. In addition, recombination is rampant within HIV populations, and several reports suggest that it may be an even more powerful force in shaping HIV evolutionary patterns than mutation. Rates approaching 10 crossovers per replication cycle have been observed within *in vitro* systems [2]. The true magnitude of these evolutionary processes becomes apparent when discussed in the context of HIV population biology. Stochastic models suggest that 10^10^ viral particles are produced each day within an infected individual, and generation time is in the neighborhood of 1.8 days [3]. This rate of production and turnover coupled with the aforementioned rates of recombination and mutation allow the virus to explore vast reaches of sequence space in short periods of time.

The evolutionary wizardry of HIV-1 comes at a substantial cost to the virus. The majority of viral particles are believed to be non-infectious due to genetic anomalies and assembly defects, reflecting the haphazard nature of the replication process [4]. Multiple reports suggest that the HIV-1 proviral DNA population in infected individuals is primarily composed of heavily mutated, replication incompetent genomes [5,6]. The mutation rate of HIV-1 may in fact walk a very narrow line between requisite evolvability (to avoid annihilation in a highly dynamic and treacherous environment) and requisite fidelity (to avoid population collapse resulting from a surfeit of deleterious mutations). Therefore, it has been hypothesized that even a marginal increase in the mutation rate of HIV-1 will result in genetic meltdown of the viral quasispecies, a phenomenon known as "error catastrophe" [7,8]. The induction of error catastrophe as an antiviral strategy has been explored extensively in the laboratory, but as of yet, this "lethal mutagenesis" approach has not been utilized in a clinical setting to manage HIV infection [9,10]. The treatment of chronic hepatitis C virus (HCV) infection with the ribonucleoside ribavirin may be an example of lethal mutagenesis [11], although the extensively characterized immunomodulatory activity of ribavirin suggests that non-mutagenic mechanisms likely contribute to its antiviral potency [12]. Nevertheless, there is an undeniable musicality associated with transforming the virus' greatest strength into its Achilles heel, and there is a pronounced need for novel therapeutic strategies due to resistance and toxicity issues surrounding existing antiretroviral agents.

A recent major development in the HIV research world involving an endogenous host-encoded mutagen has brought the concept of lethal mutagenesis to center stage. Apolipoprotein B mRNA editing enzyme, catalytic polypeptide-like 3G (APOBEC3G) was found to serve as an innate antiviral defense mechanism by introducing C-to-U changes in the minus strand DNA of retroviruses during replication (resulting in G-to-A mutations in the genomic sense strand sequence) [13]. Soon thereafter, surveys of the entire APOBEC cytidine deaminase family revealed that another member, APOBEC3F, exhibits similar antiviral potency and cDNA editing capacities [14]. The HIV-1 genome, however, encodes the 23 kilodalton protein Vif (virion infectivity factor) which specifically counteracts this defense by promoting the proteolytic degradation of APOBEC3G and APOBEC3F in the host cell (and perhaps by inhibiting the translation of these host factors as well) [15-17]. In the absence of Vif expression, APOBEC3 is incorporated into virions and the viral genome undergoes extensive G-to-A mutation, or "hypermutation", typically rendering it non-viable within a single replicative cycle. Consequently, Vif is emerging as an attractive target for pharmacological intervention and therapeutic vaccination [18-21].

**Figure 1:**
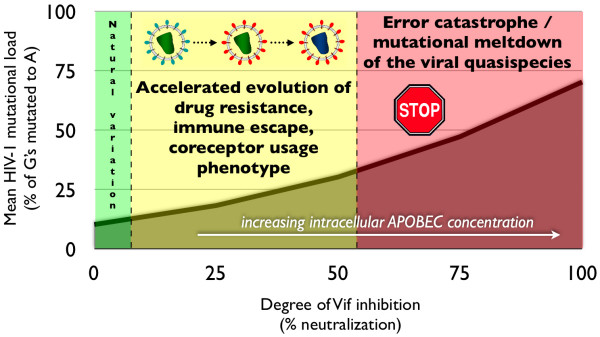
**Potential consequences of a hypothetical HIV-1 Vif-based intervention**. A highly potent Vif antagonist should significantly elevate intracellular concentrations of APOBEC3, inducing the desired end goal of viral error catastrophe (red zone). A moderately effective Vif inhibitor may fail to induce mutational meltdown and instead accelerate evolution of drug resistance, immune escape, and coreceptor phenotype (yellow zone). A weak inhibitor should invoke a level of mutational pressure that falls into the range of natural variation resulting from genetic polymorphisms in Vif and the APOBEC3 loci (green zone).

Our experience with current antiretrovirals suggests that a Vif antagonist will most likely be less than 100% effective at suppressing Vif activity, due to pharmacokinetic limitations, the presence of anatomic compartments with poor drug penetration characteristics (e.g. central nervous system), and the seemingly inevitable evolution of partial drug resistance [22]. Therefore, the possibility exists that incomplete Vif suppression will elevate intracellular APOBEC3 concentrations just enough to exert an intermediate (sub-error catastrophe) level of mutational pressure on the HIV-1 genome, accelerating viral evolution without inducing population collapse (Fig. 1). This scenario is supported by features of the HIV-APOBEC3 interaction which have been elucidated by careful and detailed experimental work:

### There is a dose response between intracellular APOBEC concentrations and degree of viral hypermutation

Based on data from *in vitro *cytidine deaminase assays, the extent of hypermutation (average number of G-to-A mutations per target sequence) is directly proportional to cellular APOBEC3G concentration [23]. If hypermutation was an all-or-nothing binary phenomenon, and APOBEC levels were only correlated with the overall number of viral genomes that were subject to hypermutation, there would be little cause for concern. However, experimental data demonstrate convincingly that both the overall number of sequences and the number of deaminated sites within each sequence scale with APOBEC concentration. Analysis of HIV-1 sequence data from infected individuals provides additional circumstantial evidence of an APOBEC dose effect. Complete genome sequences labeled as "hypermutants" (a designation which is rather arbitrary at this point) in the Los Alamos HIV Database vary quite dramatically in apparent levels of G-to-A substitution, both between and within viruses [24]. Some hypermutants are littered with canonical APOBEC-induced mutations and appear to be evolutionary dead ends, while others are barely distinguishable from non-hypermutant control sequences and may in fact represent replication competent viruses. The APOBEC dose effect is also apparent within each individual hypermutant sequence; there is a pronounced 5' to 3' mutational gradient, reflecting the length of time that the single-stranded DNA intermediate is exposed and accessible to deaminase activity during the reverse transcription process [24,25]. These observations are complemented and reinforced by the apparent stoichiometry of APOBEC3G in virions. Each delta-vif virion is believed to incorporate between 3–11 molecules of APOBEC3G [26], dependent on the presence of viral genomic RNA in the forming virion [27], and varying in tandem with intracellular concentrations of APOBEC3G in the producer cell [26]. Multiple reports demonstrate that APOBEC3G is catalytically active and has antiviral potency as a monomer. The monomeric capacity of APOBEC3G makes the range of 3–11 molecules per virion meaningful, and likely consequential [28,29].

### HIV-1 can tolerate an elevated mutation rate, and a true upper limit (error or extinction threshold) is as yet undetermined

Ample evidence suggests that HIV-1 populations may be able to withstand substantial increases in mutation rate without experiencing complete genetic meltdown. For example, AZT and 3TC treatment, as well as the mutations in reverse transcriptase that confer resistance to AZT, increase the HIV-1 error rate *in vivo *by several-fold without extinguishing the virus in the near-term. Replication of AZT-resistant viruses *in vitro *in the presence of agents like hydroxyurea and thymidine that create imbalances in deoxynucleoside triphosphate (dNTP) pools similarly increases mutation rate without destroying viability [30]. Although numerous mutagenesis studies describe significant decreases in viral fitness, titre, or infectivity as a result of mutational burden, it is unclear if these data legitimately embody the precise mathematical prerequisites of impending error catastrophe [8,10,31,32]. More importantly, extinction – that is, a detectable end to HIV replication – is rarely reported as an outcome of these experiments.

**Figure 2:**
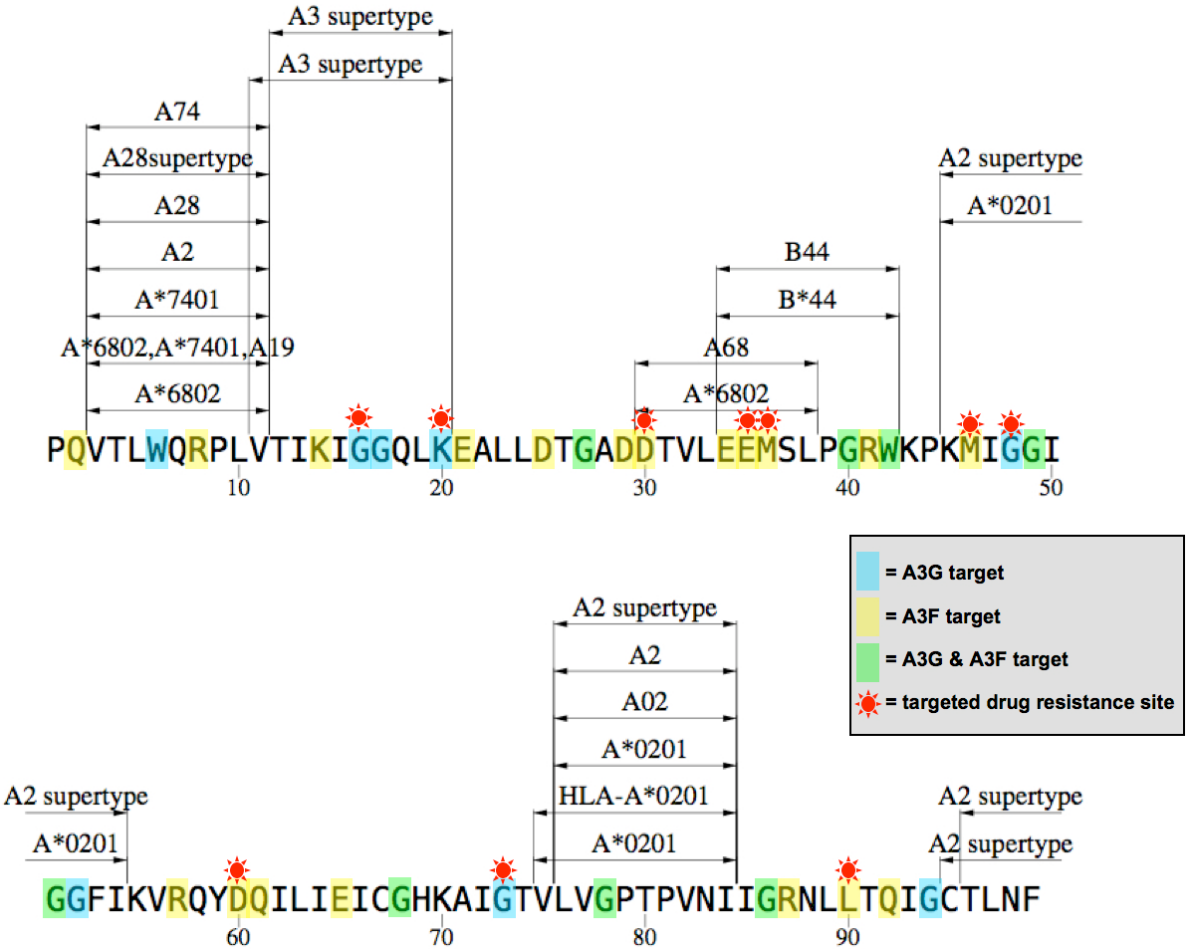
**Potential targets of APOBEC3G and APOBEC3F cytidine deaminase activity within HIV-1 protease**. Positions highlighted in blue, yellow, and green are encoded by codons that are targets of APOBEC3G, APOBEC3F, and both factors, respectively. Red stars indicate targeted positions involved in antiretroviral resistance [35]. Recognized CTL epitopes and the HLA alleles that are believed to target these regions are listed (based on prediction algorithms and/or direct experimental validation, as presented in the HIV Molecular Immunology Database [40]).

Incomplete Vif inhibition and the resulting enhancement of APOBEC3-mediated mutational pressure on the HIV-1 genome may accelerate evolution of antiretroviral resistance [33], immune escape, and cellular tropism (coreceptor usage phenotype). We can initially estimate the likelihood of these outcomes by considering the codon structure of HIV-1 and the distribution of APOBEC3G and APOBEC3F target sequence motifs across the HIV-1 proteome. APOBEC3G and APOBEC3F principally target "GG" (GG->AG) and "GA" (GA->AA) dinucleotides, respectively [25,34]. Let us consider the HIV-1 subtype B Protease consensus sequence as an example (Fig. 2). 35 of the 99 amino acids in Protease are typically encoded by codons that contain one or both of these dinucleotide target motifs. Of these 35 APOBEC3-susceptible amino acids, 19 are contained within recognized cytotoxic T cell (CTL) epitope sequences, and 10 are sites involved in resistance against protease inhibitor drugs [35]. Even when focusing on the frequency and distribution of APOBEC3 target motifs within a single HIV-1 protein, it is readily apparent that a partial Vif phenotype is likely to modulate viral susceptibility to host immunity and drug pressure.

We extended our thought experiment on Protease by performing an *in silico *exercise to investigate the relationship between enhanced APOBEC3-mediated mutational pressure and evolution of antiviral resistance. We implemented forward simulations of HIV-1 *pol *evolution under varying APOBEC3-induced mutation rates (coded in SAS version 9.1), focusing on the emergence of amino acid replacements at established protease inhibitor resistance sites [35]. Simulations were seeded with 10,000 copies of an HIV-1 consensus B *pol *sequence derived from all available subtype B data in the Los Alamos Database. Background evolution and diversification was modeled by applying a base mutation rate of 3.4*10^-5 ^substitutions/site/cycle [1] to a full 12-term substitution rate matrix inferred from a phylogeny of intrapatient *pol *sequences. APOBEC3G and APOBEC3F target dinucleotide motifs were mutated randomly each cycle at rates from 0.0001 to 0.05 (GG -> AG, GA -> AA). Sequences with premature stop codons were removed from the population at the end of each generation. Simulations were allowed to run up to 200 generations (approximately 1 year assuming an HIV-1 generation time of ~1.8 days) or until natural extinction due to depletion of viable sequences.

Rates of population extinction in our simulations varied tremendously between and even within APOBEC3 mutation rates, due to the stochastic distribution of mutations across sites and the fact that only one single codon ("TGG") at position 42 of the protease sequence is converted to a stop codon by APOBEC3 editing. Protease inhibitor resistance mutations were observed most frequently when APOBEC3G and APOBEC3F dinucleotide target motifs were mutated at rates of 0.001 to 0.01. Below a rate of 0.001, mutational patterns essentially reflected the background evolutionary model, and above 0.01, populations tended to collapse rapidly due to the accrual of stop codons. These results probably underestimate the potential for APOBEC-driven resistance evolution, due to the absence of recombination and drug-mediated positive selection pressure in our model [36-38]. Recombination should increase the sustainability of heavily mutated populations by providing a means of purging stop codons and escaping the irreversible accumulation of deleterious mutations associated with asexual reproduction known as Muller's ratchet [39]. Emulation of the selective environment associated with drug treatment (accounting for the selective advantage conferred by Protease inhibitor resistance mutations) would favor the maintenance and fixation of polymorphisms that reduce drug susceptibility. Nonetheless, this reductionist modeling exercise reinforces the notion that a highly effective Vif inhibitor may result in mutational meltdown or extinction of the HIV-1 quasispecies, while a weaker inhibitor may accelerate evolution of drug resistance and other undesirable viral phenotypic characteristics.

**Figure 3:**
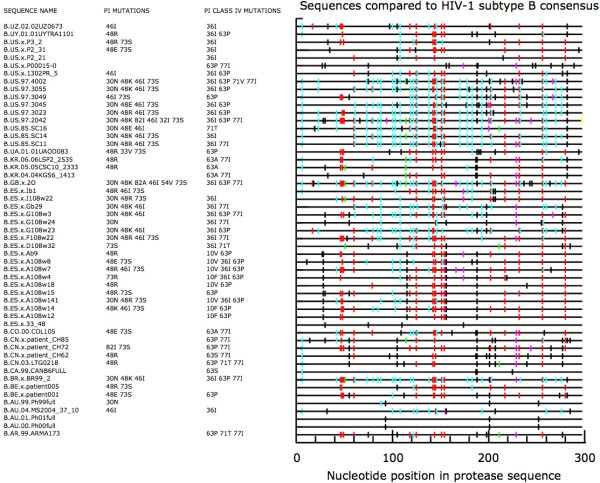
**Mutational spectra of subtype B patient-derived hypermutant HIV-1 protease sequences**. All available subtype B protease sequences in the Los Alamos Database are included, excluding experimentally generated hypermutants. When multiple identical hypermutant sequences were cataloged for a given individual, a single representative is included. Mutations occurring at recognized protease inhibitor resistance positions are listed, based on Stanford HIV Drug Resistance Database conventions [51]. Class IV mutations are listed separately, and typically need to be coupled with other mutations to contribute to resistance. The right panel displays all mutations in each sequence with respect to the subtype B protease consensus sequence (generated using Hypermut [40]); red = GG -> AG (APOBEC3G pattern), cyan = GA -> AA (APOBEC3F pattern), green = GC -> AC, magenta = GT -> AT, black = all other mutations, yellow = deletion.

The results of our simulation are mirrored in naturally occurring HIV-1 protease sequences in the Los Alamos Database [40]. A survey of all available patient-derived subtype B protease sequences from documented hypermutant genomes demonstrates that mutations at recognized protease inhibitor resistance positions are ubiquitous (Fig. 3). Nearly all of the hypermutant protease sequences have multiple non-synonymous mutations at resistance codons, all of which result from G-to-A substitutions (many of these amino acid replacements are not typically associated with resistance, but variation at these sites may impact mutational pathways and modulate viral susceptibility to antiretroviral agents to some degree). Several of the severely hypermutated sequences, however, contain a single premature stop codon at position 42 and most likely represent non-functional proteins. Such non-functional gene variants in the proviral population may still contribute to the mean fitness and stability of the HIV-1 quasispecies by serving as a repository of genetic information, available for future recombination with replication competent genomes. The array of observed nucleotide mutations confirms earlier observations that hypermutants exhibit a wide range of APOBEC3 editing intensities [24,41]; a few of the hypermutant genomes contain zero G-to-A substitutions in protease, while others have up to fifty (~75% of available G's mutated to A). Certain positions tend to be targeted across genomes and may represent previously described mutational "hotspots", where upstream nucleotides at the -1 and -2 positions favor cytidine deaminase activity [25]. Interestingly, some sequences appear to be mainly affected by APOBEC3F deaminase activity (cyan hash marks), while others are principally targeted and edited by APOBEC3G (red marks), suggesting that these mutational pressures vary independently in nature. These data demonstrate that moderately hypermutated sequences that have been subjected to intermediate levels of APOBEC3 pressure exist naturally, and moreover, these sequences often possess multiple mutations of pharmacological and immunological significance. An additional summary of observed APOBEC3-induced mutations at recognized resistance sites in HIV-1 reverse transcriptase is provided in Table 1.

**Table 1:** Observed APOBEC3-induced mutations at recognized HIV-1 RT resistance sites^a^

**HIV-1 RT Resistance Position**	**Wildtype Codon**	**APOBEC3G Target**	**APOBEC3F Target**	**Observed Mutant Codon**	**Mutant Frequency**
**M41 [NRTI]**	ATG(G)	+	-	ATA	0.7
**D67 [NRTI]**	GAC	-	+	na	0
**Q151 [NRTI]**	CAG(G)	+	-	CAA×	0.5
**M184 [NRTI]**	ATG(G)	+	-	ATA*	0.6
**L210 [NRTI]**	TTG(A)	-	+	TTA×	0.1
**E138 [NNRTI]**	GAG(A)	-	+	AAG*	0.3
**G190 [NNRTI]**	GGA	+	+	AGA	0.5
**M230 [NNRTI]**	ATG(G)	+	-	ATA	0.7

^a^All available hypermutant HIV-1 subtype B reverse transcriptase sequences in the Los Alamos HIV Sequence Database [40] were analyzed (1 sequence sampled per individual, 10 total). Listed resistance positions are potential targets of APOBEC3G and/or APOBEC3F. Class of antiretroviral resistance listed in brackets (NRTI = nucleoside RT inhibitor, NNRTI = non-nucleoside RT inhibitor) [51]. Nucleotides in parentheses are immediately downstream of codons involved in antiretroviral resistance, and are included in the APOBEC3 dinucleotide target motifs. x = synonymous (silent) mutation, * = canonical resistance codon.

Perhaps the most provocative empirical evidence that the HIV-1 quasispecies may benefit from enhanced APOBEC-mediated mutational pressure is the prevalence of naturally occurring partially defective Vif variants. Patient-derived Vif variants are frequently deficient in their abilities to neutralize APOBEC3G and/or APOBEC3F, and viral sequences from these individuals exhibit mutational profiles that are consistent with their respective susceptibilities to these host factors [41,42]. Many of the observed Vif mutations affecting function are themselves the result of G-to-A transitions within canonical APOBEC3 dinucleotide motifs, suggestive of a feedback loop between Vif sequence and APOBEC3 editing [42]. The overall highly adenine-biased base composition and codon usage of HIV-1 may in fact reflect adaptation to APOBEC-mediated mutational pressure [25]. These observations are complemented by population-level analyses of APOBEC3 expression and an unexpected detail regarding a natural polymorphism in the APOBEC3G locus. There does not appear to be any correlation between levels of APOBEC3 expression (mRNA) in peripheral blood mononuclear cells and HIV viral loads or CD4+ counts in untreated HIV-infected individuals [43], although a more rigorous study design may be required to adequately address this relationship [44]. The H186R APOBEC3G allelic variant is associated with accelerated HIV disease progression, and actually appears to have slightly *increased *antiviral potency based on *in vitro *infectivity data (cytidine deaminase activity was not directly measured) [45]. Taken together, these data strongly suggest that the HIV-1 quasispecies conveniently exploits APOBEC3G and APOBEC3F as engines of genetic diversification. The virus essentially uses Vif sequence as a volume knob to modulate its susceptibility to cytidine deaminase activity in the host, analogous to the role of mutator alleles in bacterial populations.

In conclusion, given the dim prospects for an HIV vaccine in the near future [46], complete or near complete neutralization of Vif activity is a promising therapeutic strategy that may provide a much-needed alternative to existing treatment options. However, there are clues scattered throughout the literature that therapeutic strategies targeting the Vif-APOBEC interaction may benefit virus rather than host, and therefore should not be executed without the greatest of caution and forethought. Inadvertent effects of partial Vif inhibition could include the development of drug resistance to other members of an antiretroviral regimen, or loss of immune control due to accelerated evolution of epitope escape mutations. The potential outcome of a Vif-based intervention should be examined systematically and rigorously *in vitro* and *in silico* prior to clinical deployment. Long-term serial passaging of HIV-1 in the presence of scaled APOBEC3 concentrations using variable population sizes, cellular activation states, and environmental challenges (e.g. drug exposure) should yield invaluable insight into the likelihood of treatment success. In addition, the effects of a Vif-based intervention should be explored virtually by implementing a sophisticated simulation that incorporates recombination, positive selection, and realistic population dynamics into the underlying model. On a different note, there are emerging subtleties and complexities associated with the HIV-APOBEC3 relationship that may open up additional avenues for therapeutic intervention that fall outside of the lethal mutagenesis realm. Antiviral potency has recently been attributed to both APOBEC3G and APOBEC3F that is distinct and separable from cytidine deaminase activity [47-49], although the legitimacy of this capacity is still a topic of debate [50]. A therapeutic scheme that selectively amplifies this non-mutagenic antiviral phenotype without enhancing cytidine deamination may be an effective alternate route to suppression of viral replication.

## Authors' contributions

SKP wrote the paper, conducted background research, and performed HIV sequence analysis. JKW contributed to writing and editing. JDB programmed the evolutionary simulations and contributed to writing and editing.
